# News Coverage of Colorectal Cancer on Google News: Descriptive Study

**DOI:** 10.2196/39180

**Published:** 2022-06-15

**Authors:** Corey H Basch, Grace C Hillyer, Erin T Jacques

**Affiliations:** 1 Department of Public Health William Paterson University Wayne, NJ United States; 2 Department of Epidemiology Mailman School of Public Health Columbia University New York, NY United States; 3 Department of Health & Human Performance York College City University of New York Queens, NY United States

**Keywords:** colorectal cancer, internet, online news, screening, disparities, infodemiology, online health information, content analysis, public awareness, health news, cancer screening, health video, video content analysis

## Abstract

**Background:**

Colorectal cancer (CRC) is one of the leading causes of cancer death in the United States. The incidence and prevalence of CRC have historically increased with age. Although rates of CRC in the United States have been decreasing over the past decades among those aged ≥65 years, there has been an uptick among those in younger age brackets. Google News is one of the biggest traffic drivers to top news sites. It aggregates and shares news highlights from multiple sources worldwide and organizes them by content type. Despite the widespread use of Google News, research is lacking on the type of CRC content represented in this news source.

**Objective:**

The purpose of this study was to analyze content related to CRC screening and prevention in Google News articles published during National Colorectal Cancer Awareness Month (March 2022).

**Methods:**

Data collection for this cross-sectional study was conducted in March 2022—National Colorectal Cancer Awareness Month. Using the term *colorectal cancer*, 100 English-language Google News articles were extracted and coded for content. A combined approach—deductive and inductive coding—was utilized. Descriptive analyses were conducted, and frequency distributions were reported. Univariable analyses were performed to assess differences between articles that mentioned CRC screening and those that did not via chi-square tests.

**Results:**

Of the 100 articles reviewed, nearly half (n=49, 49%) were created by health news organizations, and another 27% (n=27) were created by television news services. The predominant themes in the content included age at the onset of disease (n=59, 59%), mortality related to CRC (n=57, 57%), and the severity of disease (n=50, 50%). Only 18% (n=18) of articles discussed CRC disparities, 23% (n=23) mentioned that there are hereditary forms of the disease, 36% (n=36) spoke of colonoscopy to screen for the disease, and 37% (n=37) mentioned how the disease is treated. Although most articles mentioned CRC screening (n=61, 61%), it was striking that sex was only mentioned in 34% (21/61) of these articles, colonoscopy was mentioned in 46% (28/61), and diet was mentioned in 30% (18/61).

**Conclusions:**

Heightening the public’s awareness of this disease is important, but it is critical that messages related to how preventable this cancer is, who is the most likely to develop CRC, and what can be done to detect it in the early stages when the disease is the most curable be the critical elements of dialogue, particularly during National Colorectal Cancer Awareness Month. There is a need to disseminate information about early-onset CRC and the importance of screening, especially among populations with low rates of uptake. Web-based news is potentially an underutilized communication mechanism for promoting CRC screenings as secondary prevention measures for high-risk groups.

## Introduction

Colorectal cancer (CRC) is one of the leading causes of cancer death in the United States [1]. It is estimated that in 2022, 151,030 adults in the United States will be diagnosed with CRC, resulting in 52,580 deaths [1]. Risk factors for CRC include family history and lifestyle factors such as physical inactivity, dietary factors, overweight and obesity, and alcohol and tobacco use [2]. Emerging research also suggests that disruptions to the gut microbiome due to oral antibiotic use [3] and other factors [4-6] related to gut microbiota can also be contributing factors.

The incidence and prevalence of CRC have historically increased with age. According to recent figures, the median age of diagnosis is 69 years in women and 66 years in men [7]. Although rates of CRC in the United States have been decreasing over the past decades among those aged ≥65 years, there has been an uptick among those in younger age brackets [7,8]. According to researchers, the “incidence of colorectal cancer (specifically adenocarcinoma) in adults aged 40 to 49 years has increased by almost 15% from 2000-2002 to 2014-2016” [9]. It is important to note that not only is the incidence of CRC rising in younger Americans, but the rate of mortality is as well [10]. The US Preventive Services Task Force lowered the recommended age for CRC screening from 50 years to 45 years in 2021 [9].

Screening for CRC can drastically reduce morbidity and mortality. The US Preventive Services Task Force recommends stool-based and direct visualization–based (eg, colonoscopy, flexible sigmoidoscopy, and computed tomography colonography) tests for screening [10]. Data from the Behavioral Risk Factor Surveillance Survey indicate that 71.6% of adults aged 50 to 75 years were up-to-date with all CRC screening test types, and 69.7% were up-to-date based on the receipt of a fecal immunochemical test, sigmoidoscopy, or colonoscopy [11]. These numbers do not reflect the 2021 changes that were made to lower the recommended age for screening [11].

The efforts that have been made to increase screening for CRC have included interventions, outreach, and targeted programming, with many aiming to increase knowledge and awareness. Variations in screening uptake among Hispanic [12] and Black [13] individuals are influenced by access to health care [14] and low health literacy [15]. Understudied but important factors that can contribute to behavior change are historical factors. Oftentimes, the most notable historical factors pertain to celebrities’ engagement with a health topic [16-18]. With regard to CRC specifically, researchers noted an increase in CRC screening after a newscaster, Katie Couric, announced her CRC awareness campaign in March 2000 on the *Today Show* [19]. More recently, the untimely and tragic death of Chadwick Boseman at the age of 43 has helped to raise awareness about important issues related to CRC morbidity and mortality, specifically early-onset CRC and related CRC health disparities [20].

Researchers have reported on Google News coverage of the recent update to CRC screening recommendations [21]. Others have reported on screening changes covered in web-based newspapers [22], yet research on the extent to which Google News is used to share general CRC information is unknown. News coverage is an important channel that is used by the public to gather health information [23]. Most Americans have shifted their news consumption methods from using print, television, and radio sources to using the internet on digital devices [24]. Google News—an aggregate news hub—captures cancer-related information that is regularly consumed by the general public. Americans search for cancer-related information through search engines [25] that funnel their attention to health information sites that are often reflected in Google News [26].

Google News is one of the biggest traffic drivers to top news sites [27]. It aggregates and shares news highlights from multiple sources worldwide and organizes them by content type [28]. Despite the widespread use of Google News, research is lacking on the type of CRC content represented in this news source. The purpose of this study was to analyze content related to CRC screening and prevention in Google News articles published during National Colorectal Cancer Awareness Month (March 2022).

## Methods

### Study Design

Data collection for this cross-sectional study was conducted in March 2022—National Colorectal Cancer Awareness Month. A search using the term *colorectal cancer* yielded 100 recent Google News articles. Relevance was determined based on mentions of *colorectal cancer* and the verification that content was related to CRC in some way. All 100 articles were deemed to be relevant. The publication dates ranged between June 2021 and March 2022. The articles included for analysis were written in English and had to mention *colorectal cancer* or a known variation (ie, colon, rectal or bowel cancer). The metadata (URLs, creation dates, and categories selected) for the 100 news articles chosen for inclusion were captured and organized in Microsoft Excel. Any duplicate news articles were excluded.

A combined approach—deductive and inductive coding—was used by a single coder (ETJ). The National Cancer Institute web page [29] and the researchers' interests guided the selection of the predefined variables related to the articles that were coded deductively. Inductive coding commenced while data were analyzed as new themes emerged. Codes included descriptive information, such as the source of the posts; whether any specific individuals were mentioned; and, if so, whether a layperson or public figure was mentioned. Further categories included CRC disease–related characteristics (disparities in outcomes, mortality, the severity of disease, the spread of cancer, treatment, and related research), CRC risk factors (family history and hereditary forms of the disease, age at onset, race, sex, and antibiotic use), and CRC prevention and risk reduction (mentions of screening, colonoscopy, diet, the fear of CRC screening, and insurance coverage and costs of screening).

Descriptive analyses were conducted, and frequency distributions were reported. Univariable analyses were performed to assess differences between articles that mentioned CRC screening and those that did not via chi-square tests. A *P* value of <.05 was considered statistically significant. All analyses were performed by using IBM SPSS version 28 (IBM Corporation).

### Ethical Considerations

This study did not include the participation of human subjects. Thus, the William Paterson University Institutional Review Board determined that this study did not meet the criteria for ethics review.

## Results

Of the 100 articles reviewed, nearly half (n=49, 49%) were created by health news organizations, and another 27% (n=27) were created by television news services (Table 1). The predominant themes in the content included age at the onset of disease (n=59, 59%), mortality related to CRC (n=57, 57%), and the severity of disease (n=50, 50%). Only 18% (n=18) discussed CRC disparities, 23% (n=23) mentioned there are hereditary forms of the disease, 36% (n=36) spoke of colonoscopy to screen for the disease, and 37% (n=37) discussed how the disease is treated. Most articles mentioned CRC screening (n=61, 61%), and when they were compared to articles that did not mention CRC screening, striking differences were observed.

Articles that mentioned CRC screening more often talked about topics related to this particular cancer, such as CRC mortality (45/61, 74% vs 12/39, 31%; *P*<.001), the severity of disease (37/61, 61% vs 13/39, 33%; *P*=.008), and the risk for CRC based on age (46/61, 75% vs 13/39, 33%; *P*<.001). Although most articles mentioned CRC screening (n=61, 61%), it was striking that sex was only mentioned in 34% (21/61) of these articles, colonoscopy was mentioned in 46% (28/61), and diet was mentioned in 30% (18/61). In articles where CRC screening was not mentioned, only the treatment of this cancer was discussed more often than in those that did mention CRC screening (19/39, 49% vs 18/61, 30%; *P*=.05).

**Table 1:** Characteristics and content of Google News articles (N=100) related to colorectal cancer (CRC; June 2021 to March 2022).

			Articles, n (%)	CRC screening mentioned, n (%)		*P* value	
				Yes (n=61)	No (n=39)		
**Article-related characteristics and content**							
	**Source of information**						.20
		Internet news	9 (9)	7 (12)	2 (5)		
		Television news	27 (27)	15 (25)	12 (31)		
		Health news organization	49 (49)	26 (43)	22 (56)		
		Consumer	1 (1)	1 (2)	0 (0)		
		Educational news	7 (7)	6 (10)	1 (3)		
		Non–health organization news	1 (1)	0 (0)	1 (3)		
		Academic journal	6 (6)	5 (8)	1 (3)		
		Government	1 (1)	1 (1.6)	0 (0)		
	**Specific person mentioned**						.36
		Yes	20 (20)	14 (23)	6 (15)		
		No	80 (80)	47 (77)	33 (85)		
	**Type of person mentioned**						.63
		Layperson	16 (80)^a^	11 (79)^b^	5 (83)^c^		
		Public figure	4 (20)^a^	3 (21)^b^	1 (17)^c^		
**CRC-related characteristics and content**							
	**Disparities in outcomes**						.11
		Yes	18 (18)	14 (23)	4 (10)		
		No	82 (82)	47 (77)	35 (90)		
	**Mortality**						<.001
		Yes	57 (57)	45 (74)	12 (31)		
		No	43 (43)	16 (26)	27 (69)		
	**Severity of disease**						.008
		Yes	50 (50)	37 (61)	13 (33)		
		No	50 (50)	24 (29)	26 (67)		
	**Spread of cancer**						.34
		Yes	23 (23)	16 (26)	7 (18)		
		No	77 (77)	45 (74)	32 (82)		
	**Treatment**						.05
		Yes	37 (37)	18 (30)	19 (49)		
		No	63 (63)	43 (71)	20 (51)		
	**Related research**						.59
		Yes	34 (4)	22 (36)	12 (31)		
		No	66 (66)	39 (64)	27 (69)		
**Risk**							
	**Family history or hereditary forms of the disease**						.34
		Yes	23 (23)	16 (26)	7 (18)		
		No	77 (77)	45 (74)	32 (82)		
	**Age**						<.001
		Yes	59 (59)	46 (75)	13 (33)		
		No	41 (41)	15 (25)	26 (67)		
	**Race**						.10
		Yes	15 (15)	12 (19)	3 (8)		
		No	85 (85)	49 (80)	26 (92)		
	**Sex**						<.001
		Yes	23 (23)	21 (34)	2 (5)		
		No	77 (77)	40 (66)	37 (95)		
	**Antibiotic use**						.15
		Yes	6 (6)	2 (3)	4 (10)		
		No	94 (94)	59 (97)	35 (90)		
**Prevention and risk reduction**							
	**Colonoscopy**						.01
		Yes	36 (36)	28 (46)	8 (21)		
		No	64 (64)	33 (54)	31 (80)		
	**Diet**						.009
		Yes	21 (21)	18 (30)	3 (8)		
		No	79 (79)	43 (71)	36 (92)		
	**Fear of screening**						.84
		Yes	3 (3)	2 (3)	1 (3)		
		No	97 (97)	59 (97)	38 (97)		
	**Insurance or cost of screening**						.06
		Yes	13 (13)	11 (18)	2 (5)		
		No	87 (87)	50 (82)	37 (95)		

^a^The denominator for this percentage is 20, per the number of articles in which a specific person was mentioned.

^b^The denominator for this percentage is 14, per the number of articles in which a specific person and CRC screening were mentioned.

^c^The denominator for this percentage is 6, per the number of articles in which a specific person was mentioned and CRC screening was not mentioned.

## Discussion

### Principal Findings

March is National Colorectal Cancer Awareness Month, and it was designated as such for the express purposes of highlighting the importance of screening for CRC and promoting healthy lifestyles to decrease one’s risk for developing CRC [30,31]. On February 28, 2022, the White House released a proclamation signed by the president of the United States to kick off the media blitz [32]. With the heightened public awareness of the disease during this month, we reviewed Google News articles related to CRC during March 2022 to evaluate the content of articles viewed by the public. Notably, only 61% (61/100) of articles mentioned CRC screening in general, and of those, less than half (28/61, 46%) mentioned colonoscopy—the most commonly used method for CRC screening in the United States [23]. As CRC is a potentially preventable cancer (ie, through the detection and removal of adenomatous polyps—a known precursor to malignancy [33]), the lack of mentions of CRC screening is of special concern. Further, with the recent focus of research on early-onset CRC following the tragic passing of Chadwick Boseman from CRC at the age of 43 [34] and the recent change in the age at which to begin CRC screening (from 50 years to 45 years) [9], it is particularly concerning that less than two-thirds (61/100, 61%) of the articles mentioned screening and screening goals were not a topic of coverage.

The results of this study also indicate that news articles aggregated by Google News did not sufficiently emphasize disparities in CRC morbidity and mortality. In fact, this was mentioned in fewer than 20% (18/100, 18%) of the articles included in our sample. Disparities among racial and ethnic minorities persist [35-38], and this is an important point that should be covered in news articles.

### Comparisons With Prior Works

The dearth of health-related Google News coverage on CRC disparities aligns with research studies that indicate that Black and Hispanic populations are less likely to be advised to undergo CRC screenings [39-41]. These results support existing research that accentuates the need for increased communication efforts as an approach to influence screening uptake. Heightening the public’s awareness of this disease is important, but it is critical that messages related to how preventable this cancer is, who is the most likely to develop CRC, and what can be done to detect it in the early stages when the disease is most curable be the critical elements of dialogue, particularly during National Colorectal Cancer Awareness Month.

### Limitations

This study is limited by the small sample size and cross-sectional design. The news that was present at the time of this study can fluctuate over time. As these data represent only 1 point in time, there is no point of comparison. The findings cannot be generalized or be considered representative of all web-based news. Further, these findings cannot be used to evaluate behavior. Future studies should determine who is accessing this information and how this does or does not influence actions.

### Conclusions

There is a need to disseminate information about early-onset CRC and the importance of screening, especially among populations with low rates of uptake. Web-based news is potentially an underutilized communication mechanism for promoting CRC screenings as secondary prevention measures for high-risk groups.

## Abbreviations

CRC: colorectal cancer

## References

[1] Colorectal cancer: Statistics. Cancer.Net.

[2] What are the risk factors for colorectal cancer?. Centers for Disease Control and Prevention.

[3] McDowell R, Perrott S, Murchie P, Cardwell C, Hughes C, Samuel L (2022). Oral antibiotic use and early-onset colorectal cancer: findings from a case-control study using a national clinical database. Br J Cancer.

[4] Wolf PG, Cowley ES, Breister A, Matatov S, Lucio L, Polak P, Ridlon JM, Gaskins HR, Anantharaman K (2022). Diversity and distribution of sulfur metabolic genes in the human gut microbiome and their association with colorectal cancer. Microbiome.

[5] Tremaroli V, Bäckhed F (2012). Functional interactions between the gut microbiota and host metabolism. Nature.

[6] Clemente JC, Ursell LK, Parfrey LW, Knight R (2012). The impact of the gut microbiota on human health: an integrative view. Cell.

[7] (2020). Colorectal cancer: Facts and figures 2020-2022. American Cancer Society.

[8] Siegel RL, Miller KD, Sauer AG, Fedewa SA, Butterly LF, Anderson JC, Cercek A, Smith RA, Jemal A (2020). Colorectal cancer statistics, 2020. CA Cancer J Clin.

[9] Davidson KW, Barry MJ, Mangione CM, Cabana M, Caughey AB, Davis EM, Donahue KE, Doubeni CA, Krist AH, Kubik M, Li L, Ogedegbe G, Owens DK, Pbert L, Silverstein M, Stevermer J, Tseng CW, Wong JB, US Preventive Services Task Force (2021). Screening for colorectal cancer: US Preventive Services Task Force recommendation statement. JAMA.

[10] Colorectal cancer in young adults. National Cancer Institute.

[11] Use of colorectal cancer screening tests: 2020 behavioral risk factor surveillance system. Centers for Disease Control and Prevention.

[12] Ou JY, Warner EL, Nam GE, Martel L, Carbajal-Salisbury S, Fuentes V, Wetter DW, Kirchhoff AC, Kepka D (2019). Colorectal cancer knowledge and screening adherence among low-income Hispanic employees. Health Educ Res.

[13] Burgess DJ, van Ryn M, Grill J, Noorbaloochi S, Griffin JM, Ricards J, Vernon SW, Fisher DA, Partin MR (2011). Presence and correlates of racial disparities in adherence to colorectal cancer screening guidelines. J Gen Intern Med.

[14] Jackson CS, Oman M, Patel AM, Vega KJ (2016). Health disparities in colorectal cancer among racial and ethnic minorities in the United States. J Gastrointest Oncol.

[15] Liu D, Schuchard H, Burston B, Yamashita T, Albert S (2021). Interventions to reduce healthcare disparities in cancer screening among minority adults: a systematic review. J Racial Ethn Health Disparities.

[16] Vos SC, Sutton J, Gibson CB, Butts CT (2019). Celebrity cancer on Twitter: Mapping a novel opportunity for cancer prevention. Cancer Control.

[17] Ayers JW, Althouse BM, Noar SM, Cohen JE (2014). Do celebrity cancer diagnoses promote primary cancer prevention?. Prev Med.

[18] Noar SM, Willoughby JF, Myrick JG, Brown J (2014). Public figure announcements about cancer and opportunities for cancer communication: a review and research agenda. Health Commun.

[19] Cram P, Fendrick AM, Inadomi J, Cowen ME, Carpenter D, Vijan S (2003). The impact of a celebrity promotional campaign on the use of colon cancer screening: the Katie Couric effect. Arch Intern Med.

[20] Naik H, Johnson MDD, Johnson MR (2021). Internet interest in colon cancer following the death of Chadwick Boseman: Infoveillance study. J Med Internet Res.

[21] Krigel A, Prasad VK, Lebwohl B (2020). News coverage of the American Cancer Society's update to colorectal cancer screening guidelines. Mayo Clin Proc.

[22] Elstad EA, Sheridan SL, Lee JGL, Rini C, Earp JA, Brewer NT (2014). Have screening harms become newsworthy? News coverage of prostate and colorectal cancer screening since the 2008 USPSTF recommendation changes. J Behav Med.

[23] Martinson BE, Hindman DB (2005). Building a health promotion agenda in local newspapers. Health Educ Res.

[24] Shearer E (2021). More than eight-in-ten Americans get news from digital devices. Pew Research Center.

[25] Bader JL, Theofanos MF (2003). Searching for cancer information on the internet: analyzing natural language search queries. J Med Internet Res.

[26] Hurley RJ, Tewksbury D (2012). News aggregation and content differences in online cancer news. J Broadcast Electron Media.

[27] Olmstead K, Mitchell A, Rosenstiel T Navigating news online: Where people go, how they get there and what lures them away. Pew Research Center.

[28] Google News - Daily headlines. Google Play.

[29] Colorectal cancer—Patient version. National Cancer Institute.

[30] Colorectal Cancer Awareness Month 2022. International Agency for Research on Cancer, World Health Organization.

[31] March is National Colorectal Cancer Awareness Month. Colorectal Cancer Alliance.

[32] (2022). A proclamation on National Colorectal Cancer Awareness Month, 2022. The White House.

[33] Mandel JS, Bond JH, Church TR, Snover DC, Bradley GM, Schuman LM, Ederer F (1993). Reducing mortality from colorectal cancer by screening for fecal occult blood. Minnesota Colon Cancer Control Study. N Engl J Med.

[34] Chiu A (2020). Chadwick Boseman’s death is making young people think about colon cancer. Here’s what to know. The Washington Post.

[35] Augustus GJ, Ellis NA (2018). Colorectal cancer disparity in African Americans: Risk ractors and carcinogenic mechanisms. Am J Pathol.

[36] Hao S, Parikh AA, Snyder RA (2022). Racial disparities in the management of locoregional colorectal cancer. Surg Oncol Clin N Am.

[37] Huguet N, Angier H, Rdesinski R, Hoopes M, Marino M, Holderness H, DeVoe JE (2019). Cervical and colorectal cancer screening prevalence before and after Affordable Care Act Medicaid expansion. Prev Med.

[38] Koblinski J, Jandova J, Nfonsam V (2018). Disparities in incidence of early- and late-onset colorectal cancer between Hispanics and Whites: A 10-year SEER database study. Am J Surg.

[39] Wallace DAC, Baltrus PT, Wallace TC, Blumenthal DS, Rust GS (2013). Black white disparities in receiving a physician recommendation for colorectal cancer screening and reasons for not undergoing screening. J Health Care Poor Underserved.

[40] Wilkins T, Gillies RA, Harbuck S, Garren J, Looney SW, Schade RR (2012). Racial disparities and barriers to colorectal cancer screening in rural areas. J Am Board Fam Med.

[41] Gonzalez JJ, Wahab A, Samalik J, Ramirez E, Saint-Phard T, Gonzalez E, Adekolujo OS (2020). Barriers and facilitators of colorectal cancer screening among a Hispanic community in Michigan. J Racial Ethn Health Disparities.

